# Neurophysiological signatures of default mode network dysfunction and cognitive decline in Alzheimer's disease

**DOI:** 10.1002/alz70856_097238

**Published:** 2025-12-24

**Authors:** Mouhsin Shafi

**Affiliations:** ^1^ Berenson‐Allen Center for Noninvasive Brain Stimulation, Beth Israel Deaconess Medical Center and Harvard Medical School, Boston, MA, USA

## Abstract

**Background:**

Neural hyper‐excitability and network dysfunction are neurophysiological hallmarks of Alzheimer's disease (AD) in animal studies, but their presence and clinical relevance in humans remain poorly understood

**Method:**

We introduce a novel perturbation‐based approach combining transcranial magnetic stimulation and electroencephalography (TMS‐EEG), alongside resting‐state EEG (rsEEG), to investigate neurophysiological basis of default mode network (DMN) dysfunction in early AD.

**Result:**

While rsEEG revealed global neural slowing and disrupted synchrony, these measures reflected widespread changes in brain neurophysiology without network‐specific insights. In contrast, TMS‐EEG identified network‐specific local hyper‐excitability in the parietal DMN and disrupted connectivity with frontal DMN regions, which uniquely predicted distinct cognitive impairments and mediated the link between structural brain integrity and cognition.

**Conclusion:**

Our findings provide critical insights into how network‐specific neurophysiological disruptions contribute to AD‐related cognitive dysfunction. Perturbation‐based assessments hold promise as novel markers of early detection, disease progression, and target engagement for disease‐modifying therapies aiming to restore abnormal neurophysiology in AD

